# Synthesis of Spherical Powder of Lead-Free BCZT Piezoceramics and Binder Jetting Additive Manufacturing of Triply Periodic Minimum Surface Lattice Structures

**DOI:** 10.3390/ma15186289

**Published:** 2022-09-09

**Authors:** Vadim Sufiiarov, Artem Kantyukov, Anatoliy Popovich, Anton Sotov

**Affiliations:** Institute of Mechanical Engineering, Materials, and Transport, Peter the Great St. Petersburg Polytechnic University, 195251 Saint Petersburg, Russia

**Keywords:** piezoceramic, binder jetting, additive manufacturing, triply periodic minimum surface, spheroidization, BCZT, piezoelectric properties, lead-free piezoceramic, piezomaterial, barium titanate

## Abstract

The article presents the results of the synthesis of lead-free piezoceramic materials (Ba_0.9_Ca_0.1_)(Ti_0.9_Zr_0.1_)O_3_ (BCZT system) in spherical powder form and their subsequent application in the binder jetting additive manufacturing process. Green models were manufactured using this powder material with binder jetting, different sintering modes were investigated, and the functional piezoelectric properties were measured. Lattice structures with triply periodic minimum surface topologies, such as Gyroid and Schwarz, were designed and manufactured. It is shown that the functional properties of lattice structures depend on the parameters of the cells and the chosen topology.

## 1. Introduction

Two important directions in additive manufacturing (AM) development are the broadening of the list of materials available for fabrication and the study of the functional characteristics of structures with complex geometric shapes (lattice structures, functionally graded materials, etc.).

Binder jetting (BJ) is one of the most flexible AM processes for operations with different classes of materials. This process is used with polymers [1,2,3], metal alloys [4,5,6,7,8,9], ceramics [10,11,12,13,14,15], and composite materials [16,17,18,19,20].

As with most AM processes, a sufficiently large number of parameters and variables affect the final quality of the products manufactured using the BJ process. The most important ones can be grouped as follows: characteristics of the initial powder material (such as particle shape and morphology, average size and particles size distribution, flowability, apparent density, wettability); characteristics of the binder (spray ability and wetting behavior, and the viscosity and volatility of binder components); printing parameters (layer thickness, binder saturation, nozzle cleaning frequency, curing time, and temperature); and product geometry features (such as small holes, wall thickness, print resolution, location relative to the movement of the printhead with nozzles, etc.) [21].

Piezoceramic materials generate an electric charge during deforming or, conversely, deform at an applied electric potential. Piezoceramic is used to manufacture elements of ultrasonic vibration sources, sensor devices, energy storage devices and actuators [22,23,24], and pressure and temperature sensors in high-frequency media [25,26]. Like most ceramic materials, piezoceramic is difficult to process mechanically [27]. Therefore, the production of non-standard complex geometries from ceramic materials may be practically impossible using traditional manufacturing methods.

Producing elements with complex shapes from piezoceramic using AM is a promising direction of investigation [28]. AM has advantages such as the absence of expensive tools, easy scalability, the possibility of implementing parts with complex shapes, a high degree of material utilization, and lower production time [29]. The use of AM for the manufacture of piezoelectric materials will expand the scope of their application and expand the possibilities of forming multi-layer structures and structures with complex geometric shapes. An increased level of freedom in the geometry of piezoelectric elements will allow for significant improvements in the characteristics of many devices based on piezoelectric and ferroelectric materials [30,31].

There are publications in the literature that demonstrate reasonably successful experiences as regards manufacturing piezoceramic elements using AM processes [13,15,32,33].

Li et al. [34] investigated manufactured barium titanate micro-spheres for the DLP 3D printing of polymer matrix piezocomposites with high dielectric permittivity. Barium titanate micro-spheres were obtained using plasma spheroidization technology. The authors showed that the obtained particles of barium titanate had a spherical shape, a smooth surface, a particle size of about 38 µm, improved mechanical strength, and high purity, which allow for the possibility of UV-curing-based 3D printing. First of all, the use of spherical powder significantly improves the fluidity and UV-curing performance of the composite material, with the elevated UV-curing depth increasing by a maximum of 542%. The authors demonstrated the results of an experiment involving 3D printing a polymer matrix piezocomposite (0–3) with enhanced dielectric properties.

Xu et al. [35] used the computer modeling method and found that using complex geometric structures with a topology of a triply periodic minimal surface (TPMS), such as Schwartz, Gyroid, and Neovius, can significantly increase the output characteristics of composite piezoelectric elements (the output voltage increases 2-to-8-fold).

There are no publications focused on the results of studies of BCZT lead-free piezoceramics spherical powder synthesis, the use of this material in the binder jetting additive manufacturing process, or experimental studies of the functional piezoelectric properties of samples with complex lattice structures. In this work, we demonstrate the possibility of spherical lead-free piezoceramic powder synthesis (Ba_0.9_Ca_0.1_)(Ti_0.9_Zr_0.1_)O_3_ for the BJ process and the functional properties achieved in 3D-printed piezoelectric elements with complex TPMS lattice structures.

## 2. Materials and Methods

The particle size distribution of the powders was determined by laser diffraction, i.e., Analysette 22 NanoTec plus (Fritsch, Idar-Oberstein, Germany), with a total measurement range of 0.01–2000 µm.

The Tescan Mira 3 LMU (TESCAN, Brno, Czech Republic) scanning electron microscope (SEM) operating at magnifications from 4× to 10^6^× with an accelerating voltage from 200 V to 30 kV was used for powder characterization.

The phase composition was analyzed using a Bruker D8 Advance X-ray (Bruker corp., Billerica, MA, USA) diffractometer (XRD) using CuKα radiation (l = 1.5418 Å).

The basic technological process for the received lead-free piezoceramic material (Ba_0.9_Ca_0.1_)(Ti_0.9_Zr_0.1_)O_3_ was solid-state synthesis. Powders of barium carbonate, titanium dioxide, calcium carbonate, and zirconium dioxide were used as starting materials.

Table 1 shows the results of the particle size distributions and phase composition measurements of the starting materials. 

The spheroidization was carried out by induction plasma treatment in the Tek-15 device (Tekna Plasma Systems Inc., Sherbrooke, QC, Canada).

The density of the sintered samples was measured using the Archimedes method, and relative density was calculated while taking into account the theoretical density of BCZT, i.e., 5.86 g/cm^3^.

Thermogravimetric analysis (TGA) and differential scanning calorimetry (DSC) were performed using a SETSYS Evolution 16 (Setaram, Caluire-et-Cuire, France).

The piezoceramic samples were manufactured on the ExOne Innovent system (The ExOne Company, North Huntingdon, PA, USA). This system relates to the BJ additive manufacturing process. The original ExOne BS004 solvent binder (ethylene glycol monobutylether-based solution) and CL001 cleaner (2-butoxyethanol-based solution) were used for the printing of the functional ceramic components. 

After the 3D printing, the platform (together with the green models in the surrounding powder) was placed in the thermal furnace (Yamato DX412C, Yamato Scientific, Santa Clara, CA, USA) at 180 °C for 3 h for curing. 

Solid-state synthesis and sintering were achieved in a high-temperature furnace (KJ-1700X, Zhengzhou Kejia Furnace Co., Ltd., Zhengzhou, China).

Discs from the powder after spheroidization were manufactured by traditional uniaxial pressing and sintering for a comparison of the functional properties with samples produced by BJ.

All samples were poled in air at 150 °С. An electric field of 0.6 kV/mm was applied to samples during heating, maintained for 3 min, and then samples were cooled to room temperature.

The capacity of the sample and the loss tangent were measured with an E7-28 immittance analyzer at a frequency of 1 kHz and an effective voltage of 0.5 V. The piezoelectric coefficient d_33_ was determined on polarized samples using the APC YE2730A setup using a quasi-static method.

## 3. Results and Discussion

### 3.1. Synthesis and Spherical Powder Production

For solid-state synthesis, all components of the starting materials were weighed according to the molar fractions of the final composition. Wet mixing was carried out using an attritor with the addition of isopropyl alcohol in a mass ratio of 1:1 to the total mass of the starting powders; ZrO_2_ balls were used in a mass ratio of 10:1 to the total mass. Mixing was carried out for 3 h with stops for 15 min after each hour. At the end of mixing, the material was unloaded, and drying (evaporation of isopropyl alcohol) was carried out at a temperature of 100 °C for at least 12 h.

**Figure 1:**
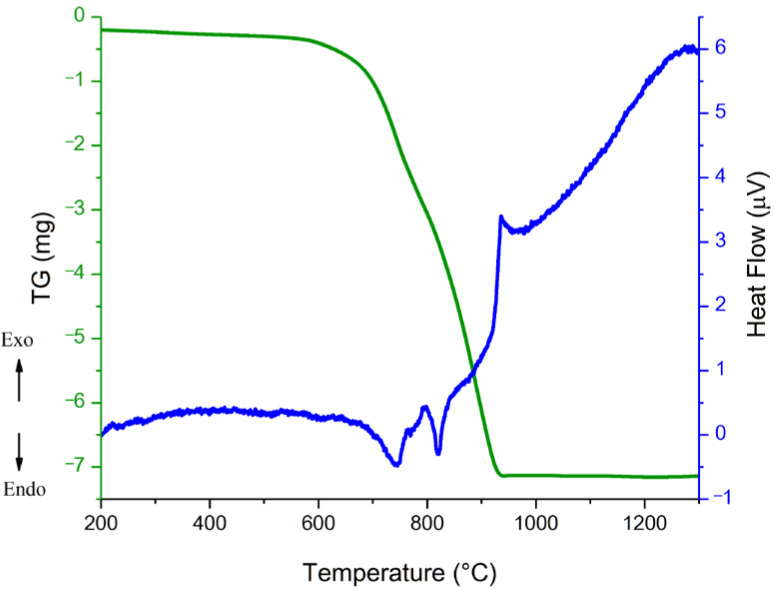
The TGA-DSC results of a powder mixture for BCZT synthesis under heating.

A thermogravimetric analysis in conjunction with differential scanning calorimetry was conducted to determine the synthesis temperature. The results are presented in Figure 1.

**Figure 2:**
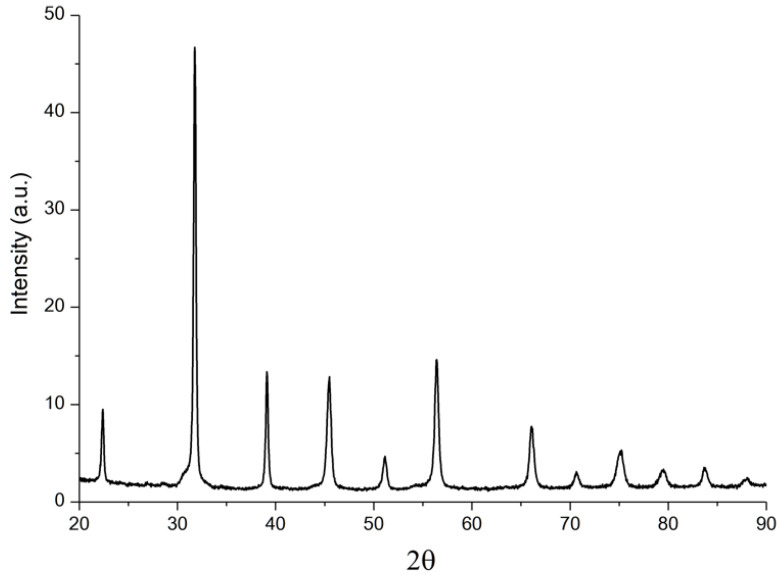
XRD results of BCZT powder after solid-state synthesis.

According to the DSC results, it can be seen that complete synthesis occurred at a temperature no lower than 1000 °C; therefore, solid-phase synthesis was carried out at a temperature of 1000 °C, with a dwell time of 2 h. After solid-state synthesis, the phase composition of the material was studied (Figure 2).

There are no peaks corresponding to the initial components (BaCO_3_, CaCO_3_, SiO_2_, and ZrO_2_) or BaO and CaO in the XRD results, which indicates the full completion of the synthesis. The phase composition of the final material was a compound of tetragonal structure based on barium titanate.

The BCZT powder obtained after solid-state synthesis had a particle size from 100 to 500 nm. However, a powder with such a particle size is usually not used in the BJ process. The BJ technology generally requires the use of a comparably larger powder, preferably spherical or rounded, with particle sizes from 3 to 150 microns. Further agglomeration by partial sintering of the powder and subsequent plasma spheroidization were carried out.

**Figure 3:**
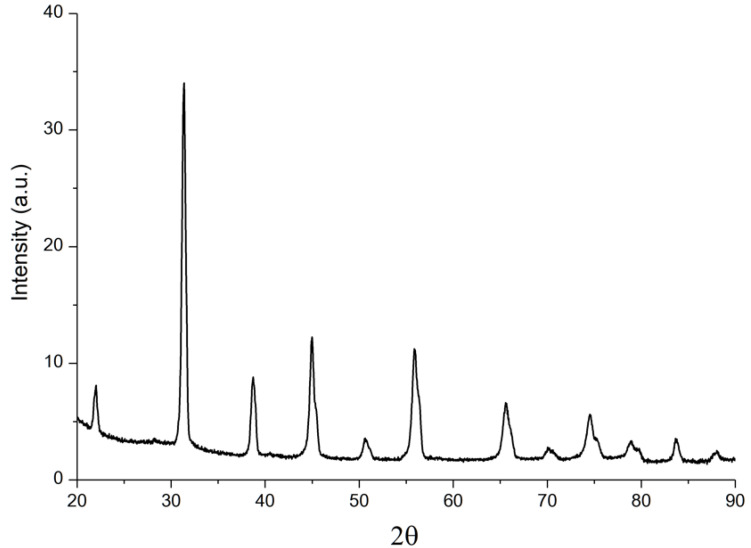
XRD results of BCZT powder after plasma spheroidization.

After plasma spheroidization, the powder material was investigated. Figure 3 shows the result of the XRD analysis. The phase composition did not change and represents a compound of tetragonal structure based on barium titanate.

**Figure 4:**
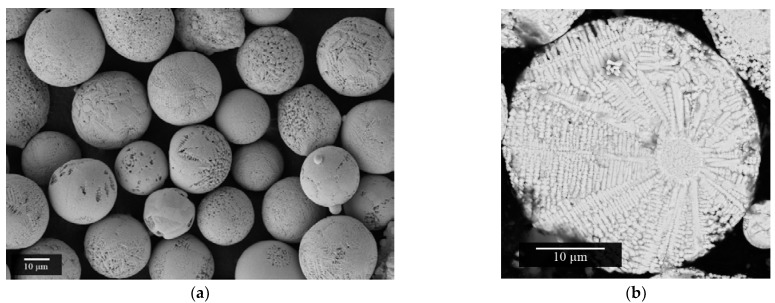
SEM images of BCZT powder after plasma spheroidization: (**a**) general view; (**b**) micro-section.

The SEM investigation (Figure 4a) indicates that powder particles predominantly possessed a spherical shape. The study of the particle micro-section showed that the internal micro-structure had a dendritic morphology (Figure 4b).

The measurement of the particle size distribution established numerical values of the main parameters: D_10_—10.3 µm, D_50_—31.8 µm, and D_90_—100.4 µm. Flowability was also measured by flowing the sample through a Hall funnel. It was 50 s/50 g with an apparent density of 2.49 g/cm^3^.

### 3.2. Binder Jetting and Post-Treatment

**Figure 5:**
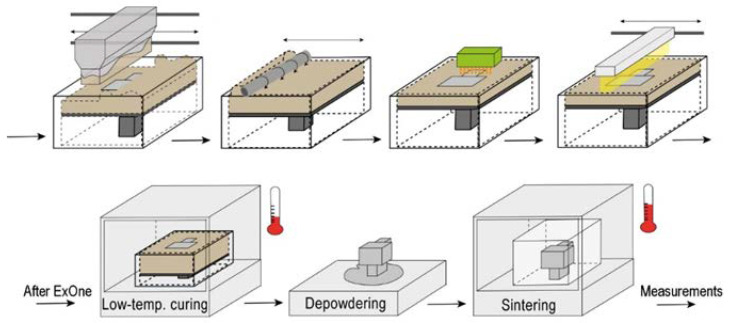
Schema of AM of piezoceramic samples based on BJ process.

Based on the results of the preliminary tests, the following modes were used as printing parameters: layer thickness: 100 µm; saturation: 80%; roller speed: 6 mm/s; roller rotation speed: 200 rpm; vibration frequency of powder material feeder: 2000 rpm; speed of powder material feeder during the formation of a layer: 42 mm/s; drying temperature: 55 °C; and drying time: 30 s. Figure 5 shows the schema of the additive manufacturing of piezoceramic samples based on the BJ process.

In the first stage, using an oscillator, the powder is fed from the feeder into the working area, and a thin layer of powder material is formed on the platform using a roller. Next, the binder is jetted from the printhead to areas of the powder layer determined by a computer model. The powder layer is heated by an infrared lamp to fix the binder. Then, the platform is lowered to a specified layer thickness and the processes described earlier are repeated. After 3D printing, the platform is placed in a thermal furnace for the complete curing of the binder. After curing, the workpieces have sufficient strength to be removed from the surrounding powder and any excess removed. A brush or air purging is used for cleaning.

**Figure 6:**
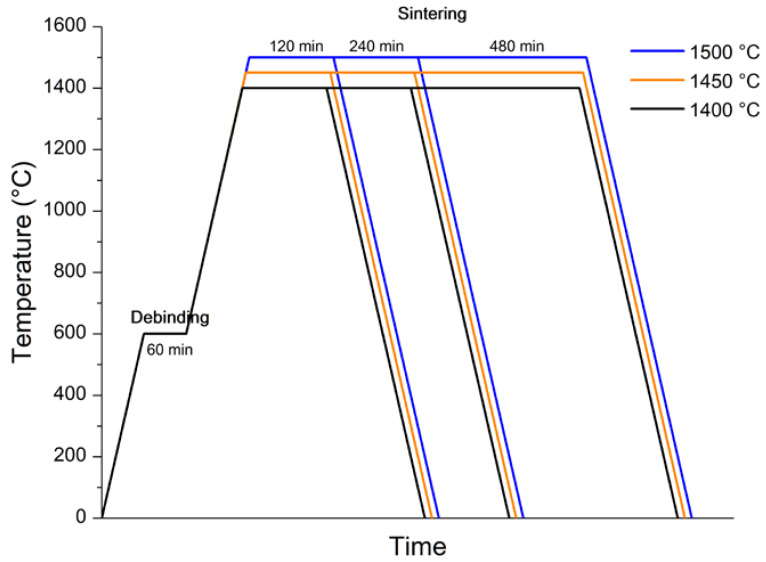
Heat treatment profiles of BCZT piezoceramic after 3D printing.

**Figure 7:**
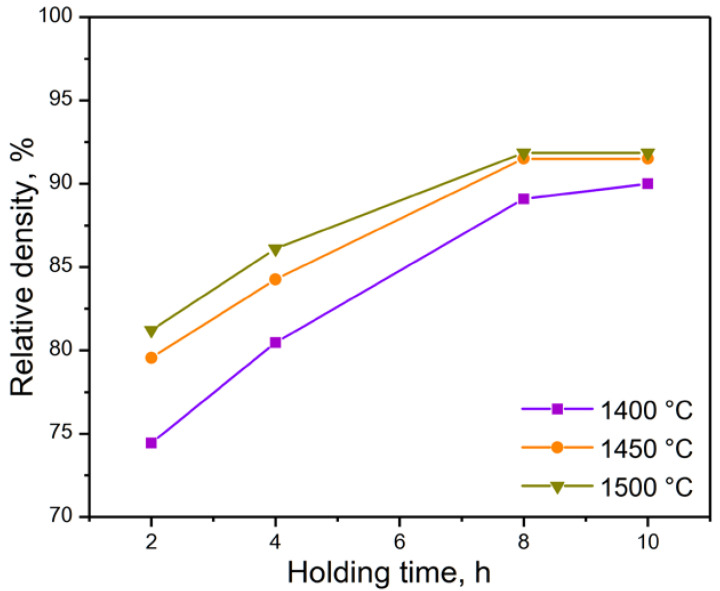
Changes in the relative density of samples after sintering at different modes.

The sintering process was carried out in two stages: first, debinding was carried out at a temperature of 600 °C, and then, the temperature was increased for sintering (a temperature from 1400 to 1500 °C; a dwell time of 2, 4, 8 and 10 h). Generalized profiles of thermal post-treatments are shown in Figure 6. A study of material densification after sintering in different modes was carried out (Figure 7).

As a result of sintering, the highest density was obtained at temperatures of 1500 °C and 1450 °C, with a dwell time of 8 and 10 h. Due to there being no significant changes in the density of the material between a dwell time of 8 and 10 h, all further experiments were conducted with samples sintered at 1500 °C and a dwell time of 8 h.

**Figure 8:**
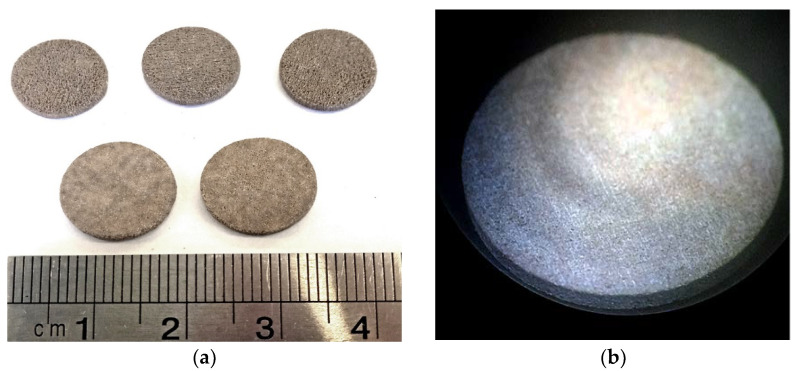
Images of BCZT samples manufactured by BJ for the functional property measurements: (**a**) samples after sintering; (**b**) samples after silver electrode coating.

To determine the functional properties of the material manufactured by BJ and subsequent sintering, cylindrical samples were manufactured, the final size of which was 10 mm in diameter and 1 mm in height (Figure 8a); sintering occurred at 1500 °C, with a dwell time of 8 h. Top and bottom surfaces were coated by silver electrodes (Figure 8b). The samples were polarized in the air at a temperature of 20 °C above the Curie temperature using an electric field of 1.6 kV/mm for 2 min.

The piezoelectric properties were measured a day after polarization. The dielectric permittivity ε_33_/ε_0_ at 1 kHz was 1909, dielectric loss tan*δ* was 1.9, the value of the piezoelectric coefficient d_33_ was 152 pC/N, and the electromechanical coupling coefficient K_p_ was 0.151. The piezoelectric properties of samples obtained using traditional piezoceramic technology using BCZT powder after plasma spheroidization were as follows: the dielectric permittivity ε_33_/ε_0_ at 1 kHz was 2229, dielectric loss tan*δ* was 0.9, the value of the piezoelectric coefficient d_3_ was 165 pC/N, and the electromechanical coupling coefficient K_p_ was 0.212. However, properties of samples obtained using AM were inferior to samples obtained using traditional piezoceramic technology. This may be explained by the non-optimal mode of binder removal and sintering, the presence of pores and, as a consequence, a decreased volume of active piezoceramic phase in the sample.

### 3.3. Additive Manufacturing of Triply Periodic Minimum Surface Lattice Structures

To investigate the possibility of manufacture and the properties of objects with a complex geometry, lattice structures with the topology of TPMS Schwarz, Neovius, and Gyroid types were designed. The cell size was chosen to be 4 mm. The experiments with varying wall thicknesses showed that the printhead resolution was limited by a wall thickness of 0.25 mm. Furthermore, Neovius-type structures were not successfully manufactured in the investigated wall-thickness ranges (up to 1 mm). Destruction of samples with thin wall thicknesses mainly occurred in the depowdering stage, with the strength of the green models not being sufficient for extracting and cleaning.

**Figure 9:**
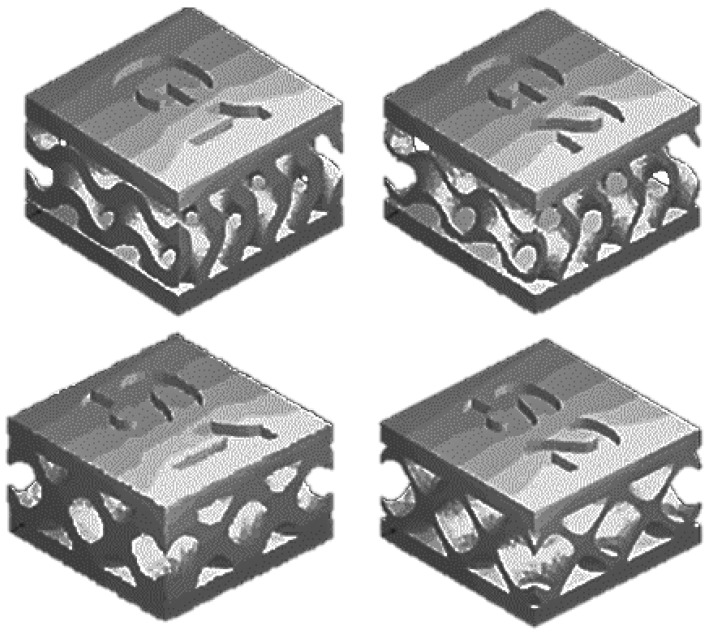
Computer models of TPMS structures of Gyroid- and Schwarz-type topology.

The geometric parameters of the TPMS samples successfully manufactured by 3D printing are presented in Table 2, and Figure 9 presents images of their computer models.

**Figure 10:**
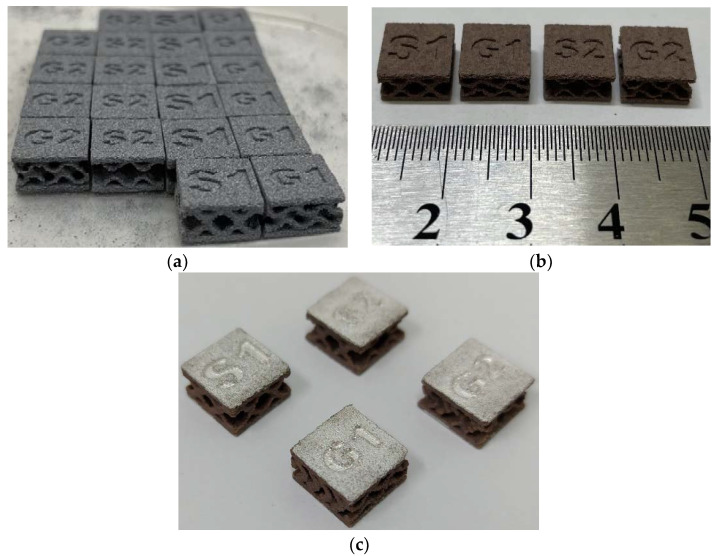
Images of TPMS structures after: (**a**) manufacturing by BJ; (**b**) sintering; (**c**) coating of electrodes for measuring the functional properties.

Sintering and polarization of TPMS samples were achieved under the same conditions as those previously described for the cylindrical samples. Figure 10 shows images of samples at different stages of manufacturing.

Piezoelectric properties TPMS samples manufactured by BJ from BCZT spheroidized powder are presented in Table 3.

Samples with thicker walls exhibited higher functional property values (only K_p_ was exceptional as regards this general trend), dielectric permittivity increased in proportion to the change in wall thickness, and the piezoelectric coefficient values became 10–20% higher with a 100% increase in the wall thickness.

The decrease in the piezoelectric coefficient d_33_ of TPMS samples, in comparison with the cylinder samples, was probably caused by a decrease in the total volume fraction of the piezoceramic material and the features of the complex ceramic architectural structure. In general, such a comparison is not completely accurate, since in the case of the cylindrical samples, the material and manufacturing technology were evaluated, and when measured on the TPMS samples, the influence of geometric features on the functional properties was additionally evaluated.

Moreover, attention should be paid to the values of the electromechanical coupling coefficient K_p_ measured for the TPMS samples. This parameter characterizes the efficiency of converting mechanical energy into electrical energy (and vice versa) and may be a quality criterion for such complex structures when measuring their functional properties. For cylindrical samples, the electromechanical coupling coefficient Kp was 0.151, whereas for all TPMS samples, Kp exceeded this value, e.g., in the case of the Gyroid-type topology, this twofold or more. This indicates a greater sensitivity and efficiency when converting mechanical action into electrical energy, which can have a positive impact on a number of potential applications of piezoelectric elements with complex lattice structures.

When comparing the results of the functional properties of the cellular structures of different topologies, it should be noted that all the values of the main characteristics were higher for structures with a Gyroid-type topology.

## 4. Conclusions

In the research presented herein, we demonstrate the possibility of applying solid-phase synthesis and subsequent plasma spheroidization for the production of promising lead-free piezoelectric (Ba_0.9_Ca_0.1_)(Ti_0.9_Zr_0.1_)O_3_ material spherical powders. Using this powder material and the binder jetting additive manufacturing process, samples were produced in order to measure the functional characteristics, i.e., the dielectric permittivity at 1 kHz was 1909, the piezoelectric coefficient was 152 pC/N, and electromechanical coupling coefficient was 0.151.

BJ technology makes it possible to produce objects with complex geometries, which was demonstrated by manufacturing lattice structures with TPMS topologies, such as Schwarz and Gyroid types. The piezoelectric properties were also measured for these structures, a distinctive feature of which was the high values of electromechanical coupling coefficient, e.g., two or more times those of the values measured for the cylindrical samples. This feature may be positive for a number of potential applications using piezoelectric elements, such as medical devices, sonar, pressure sensors, etc. The greatest effect associated with the increasing electromechanical coupling coefficient was achieved in Gyroid-type lattice structures.

## Figures and Tables

**Table 1:** Characteristics of the starting powder materials for BCZT synthesis.

Material	D_10_	D_50_	D_90_	Phase Composition
CaCO_3_	26.0 µm	50.9 µm	89.6 µm	Calcite
BaCO_3_	0.8 µm	5.0 µm	16.1 µm	Witherite
TiO_2_	204 nm	447 nm	813 nm	Rutile + Anatase
ZrO_2_	214 nm	295 nm	407 nm	Baddeleyite

**Table 2:** The geometric parameters of TPMS samples.

Lattice Type	Wall Thickness	Cell Size	Label
Schwarz	0.5 mm	4 mm	S1
Schwarz	0.25 mm	4 mm	S2
Gyroid	1 mm	4 mm	G1
Gyroid	0.5 mm	4 mm	G2

**Table 3:** Piezoelectric properties of TPMS samples.

Label	ε_33_/ε_0_	tanδ	d_33_, pC/N	K_p_
S1	404	2.8	110	0.170
S2	165	3.1	91	0.297
G1	528	3.2	113	0.564
G2	222	3.2	105	0.312
